# Course of an unplanned and unexpected pregnancy in a 39 year-old patient with Complex bladder extrophy: a case report

**DOI:** 10.1186/s13256-023-04181-9

**Published:** 2023-10-29

**Authors:** Susanne Schrey-Petersen, Martin Lacher, Holger Stepan

**Affiliations:** 1 Department of Obstetrics, University of Leipzig, Liebigstr. 20A, 04103 Leipzig, Germany; 2 Department of Pediatric Surgery, University of Leipzig, Leipzig, Germany

**Keywords:** Bladder extrophy, Pregnancy, Case report, Transfer of care, Extrophy-epispadias-complex

## Abstract

**Background:**

With improved operative techniques pregnancy rates have been rising in patients with anomalies of the extrophy-epispadias-complex, including also female patients with bladder extrophy. Specific risks around pregnancy need to be addressed sufficiently beforehand.

**Case presentation:**

An unplanned pregnancy was detected at 34 weeks in a 39-year old White female patient with former complex bladder extrophy. Decades after her operation she had not received any follow-up medical care and believed to be unable to conceive due to her anomaly. Thus no contraceptive matters were taken. The patient had lived in a stable relationship with regular sexual intercourse for many years. Until 34 weeks the pregnancy was uncomplicated, but then uterine prolapse and signs of beginning pre-eclampsia appeared, and a healthy girl was born with cesarean section.

**Conclusion:**

As patients with bladder extrophy and other anomalies from the extrophy-epispadias-complex reach adolescence/adulthood, they need continuous medical follow-up and transition of care to adult surgery and gynecology in order to address specific aspects of sexual health, reproduction, contraception, and also cancer screening. In the presented case lack of transition of care resulted in an unplanned and complicated pregnancy.

## Background

Anomalies of the extrophy-epispadias-complex comprise a wide spectrum of complex defects of the genitourinary tract, including anomalies of the abdominal wall, pelvic floor, bony pelvis and lower spine. They can be divided into three subtypes: epispadias, classical bladder extrophy, and cloacal extrophy [1], but crossovers and associated anomalies are common. Complete epispadias has an incidence of one in 117,000 male life births and one in 484,000 life births in females [1, 2]. Classical bladder extrophy has an incidence one of 10,000 to 50,000 births [1, 3] and affects males approximately twice as often as females [1, 4]. Cloacal extrophy, which is also known as OEIS complex (omphalocele-exstrophy-imperforate anus-spinal dysraphism) is much more rare: one review showed 112 patients with cloacal extrophy out of all 1202 patients with anomalies from the extrophy-epispadias complex [1, 5].

Children with all of these anomalies usually undergo multiple operations and complex repair. Over the last decades, operative techniques and results have largely improved, and besides the “classical” aspect of urinary continence, reproductive topics as sexual health, contraception and reproduction have moved into focus. As a rising number of patients with congenital anomalies reaches adulthood and reproductive age, continuation of medical care into adulthood is often a critical point with regards to necessary follow-up examinations and adequate patients counseling. We report the case of a 39-year old woman with a complex bladder extrophy, who was operated by cystectomy and continent anal urinary diversion shortly after birth, and now presented with an unexpected and unplanned 2nd trimester pregnancy.

## Case presentation

The 39-year old, white female was first seen at our clinic at 34 gestational weeks. She was born with a complex anomaly including extrophy of the bladder, anteriorly located and abnormally formed external genitalia, musculoskeletal abnormalities of the pelvis (pubic diastasis) and an abdominal wall defect (omphalocele). Shortly after birth, she was operated by cystectomy and **continent anal urinary diversion**, which means, that her ureters where implanted into the rectum (Mainz Pouch II). She reported to be continent for urine and faeces over the intact sphincter ani, and did not have any history of frequent kidney infections at any point in her lifetime. The uterus and the cervix were of normal appearance, and at time of presentation, the woman had a closed abdominal wall with a vulva-like opening in her lower abdomen, over which she had regular menstruations as well as sexual intercourse. All further physical examinations (heart, lung, skeletal) were normal. She lived in a stable partnership (married) and had a regular job as a sales attendant at a gas station. The patient reported to smoke one cigarette per day. As a young girl she was told, that she could never become pregnant, and thus she did not use any form of contraception and also never went to see a gynecologist.

**Fig. 1:**
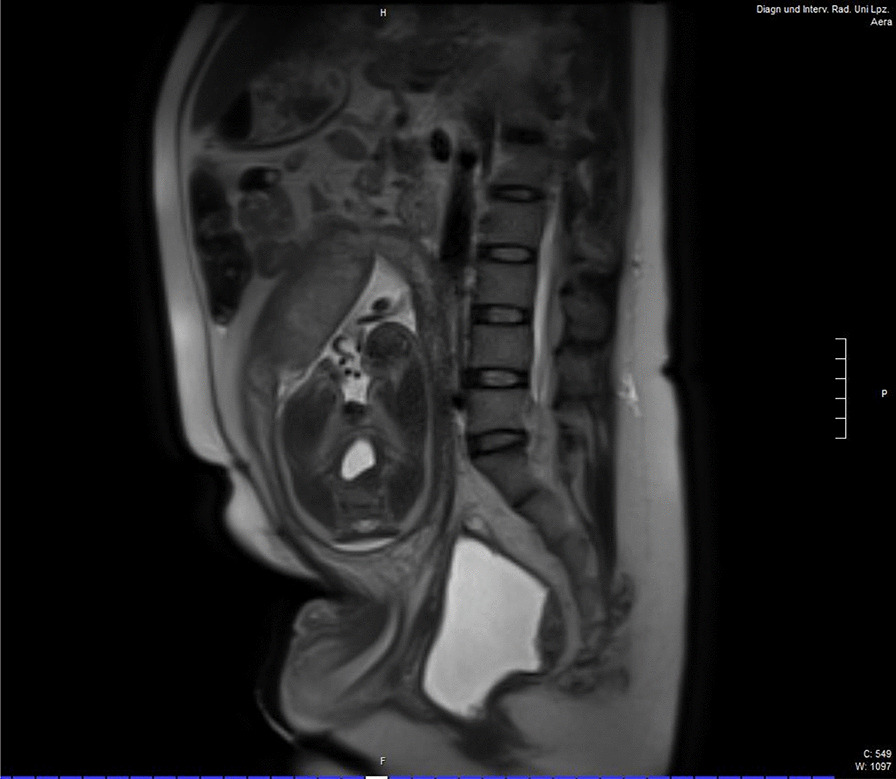
Magnetic resonance imaging (MRI) of the patient at 34 weeks gestational age showing the exact position of the uterus and the fetus in breech position

The present pregnancy was her first at 39 years of age, and was not detected until over 20 weeks. The local obstetrical team was unsure about delivery management, and thus she was referred to our tertiary care center at 34 weeks. At this point, the patient was healthy, and no anomalies were found in the fetus, and uterine and umbilical perfusions were normal. The fetus was, however, growth restricted (estimated weight below 3rd percentile). Lung maturity treatment was initiated with 2 × 12 mg betamethason, and it was planned to prolong pregnancy until 36 weeks. As mode of delivery a cesarean section over a midline laparatomy in the upper abdomen was planned, which was above the region of the previous operations. Here, the uterine wall could be well demonstrated on ultrasound a few centimeters behind the abdominal wall. Magnetic resonance imaging (MRI) (Fig. 1) was performed to localize the exact position of the ureters. There were no reports of the primary operations performed shortly after birth 39 years ago.

**Fig. 2:**
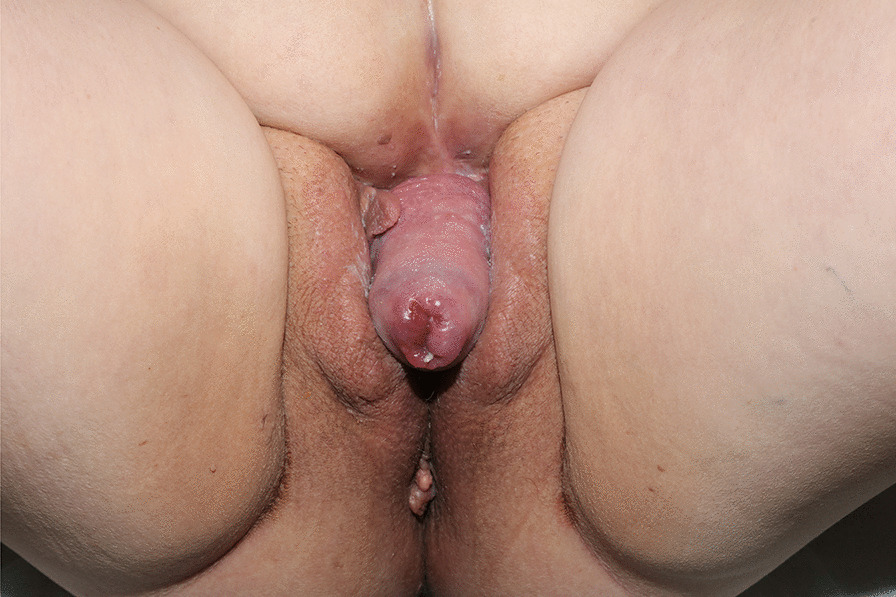
Cervical prolaps through the vaginal-like opening in the abdominal wall at 35 weeks of gestation

As the patient lived in some distance from our center, she was hospitalized at 35 weeks to wait for delivery. At this time, the cervix had prolapsed through the abdominal wall (Fig. 2). Cervical length continued to be normal, and no signs of infection were seen.

Shortly after hospitalization, the patient started to show symptoms of beginning preeclampsia (increased blood pressure with a maximum of 170/100 an elevated sFlt-1:PlGF ratio of 260, rising to 365, but no proteinuria or clinical symptoms) and non-reassuring fetal heart rate tracing (variable decelerations). Treatment was started with methyldopa 3 × 250 mg per day. Apart from nulli-parity and advanced maternal age, the patient did not have any other risk factors for preeclampsia. But fetal growth restriction can be interpreted as an additional sign of dysfunctional placenta in this patient, although uterine perfusion was always normal. Cesarean section was performed at 35 + 2 weeks and a growth restricted, but otherwise healthy preterm girl was delivered with a birth weight of 1490 g (< 3rd percentile), Apgars of 8/10/10 and an umbilical cord artery pH of 7,21. The cesarean section was uncomplicated, and no major adhesions were found in the upper abdomen. A tubal ligation was performed, as the patient did not wish any further pregnancies. The postpartal maternal course was uncomplicated with normalizing blood pressure under continuous therapy with methyldopa (3 × 250 mg per day). The patient was discharged at day 6 post cesarean and advised to see an internal medicine specialist for re-evaluation of hypertension 6 weeks postpartum. Uterine prolapse was still present at this point. The baby was discharged from the intensive care unit 16 days after delivery. The mother was advised to have regular coloscopies at least every 5 years, if an initial coloscopy showed normal findings, as continent anal urinary diversion bears an increased risk of rectal carcinoma. As the patient lived about 2 h away from the perinatal center, she wished all follow-up care to be performed closer to her home. In preparation of this case reports, it was attempted to reach the patient, but she had moved and changed her telephone number, so no information over her further follow-up could be obtained.

## Discussion

Successful pregnancies in patients with bladder extrophy or other anomalies of the extrophy-epispadias-complex are rare, but become more frequent as operative results have improved over the last decades.

In our case, this patient with bladder extrophy was operated shortly after birth with excision of the bladder plate, continent anal urinary diversion and closure of her omphalocele. This operation was standard of care until the 1970s or in some places 1980s, but was since left by centers, as it has been associated with an increased risk of malignancies originating from the rectal reservoir [6]. Nowadays, if the bladder cannot be preserved, patients would receive a “neo-bladder”, e.g. a conduit. Our patient was firstly informed of this increased risk of rectal cancer at our center and advised to have coloscopies on a regular basis in the future.

The patient had never seen a gynecologist or been counseled regarding contraception as she was told as a young girl, that she could never become pregnant. She also never attended cervical cancer screening. The counselling of the patient is in large contrast to what we now know about sexual activity and life expectancy in patients with *bladder or cloacal extrophy*: Results from a German multi-center study from 2017 show, that 81% of twenty-one patients (aged ≥ 18 years) with congenital anomalies of the extrophy-epispadias complex had sexual intercourse, and 62% thereof on a regular basis, although 19% experienced some form of pain or discomfort thereby [7]. This study matches the results from other studies, which also report a high rate of sexual activity of 89% [8] in patients with classical bladder extrophy, or a sexual function, which was better than normal [9] in patients with bladder extrophy or epispadias, although sexual activity was started later in life and patients had less children as compared to the general population. In patients with continent anal urinary diversion, a sexual activity rate of 88% was reported [6]. Patient counseling should therefore include reproductive health topics, including physical and psychological problems around sexual intercourse, contraception, cancer screening and also pregnancy including its specific risks in these patients.

Pregnancies, especially those carried out until delivery of a viable child, are rare in patients with abnormalities of the extrophy-epispadias complex. This is mostly due to the rarity of these anomalies as reported fertility rates in patients with classical bladder extrophy range between 25 and 68% [7, 8, 10–14]. In patients with continent anal urinary diversion, a pregnancy rate of 31% has been reported in one retrospective study, where 5 (of 14) women became pregnant and delivered 6 healthy children [6]. At least one case report describes a pregnancy in a woman after cloacal extrophy repair [15].

As female epispadias is a rare anomaly, no specific pregnancy rates are reported for these patients, although pregnancies are expected to be less complicated as compared to those in patients with cloacal or bladder extrophy. Most reported pregnancies in patients with bladder extrophy are received naturally [7, 14], but some authors state, that there might be a higher risk of infertility, and patients should therefore seek early consultation by a reproductive medicine specialist [14].

Pregnancies in patients with bladder extrophy, and more so in those with cloacal extrophy entail specific risks, which include prolapse of the vagina or uterus due to pelvic instability, urinary complications (hydronephrosis, pyelonephritis [16–18]) and operative risks during delivery [8]. Almost all reported patients have been delivered by cesarean section to avoid damage of the reconstructed genito-urinary tract, but severe damage has been reported even if an urologist was present during delivery [7, 8]. Mathew *et al.* reported one uneventful vaginal delivery of seven pregnancies in women with bladder extrophy [12]. In the presented case the patient developed uterine prolapse during pregnancy. Ebert *et al.* illustrated a similar case [7], and Deans *et al.* described a rate of pelvic floor prolapse of 42% (8/19), and stated that uterine prolapse during pregnancy might occur in as much as 50% of women with bladder extrophy [8]. Whether placental insufficiency, fetal growth restriction and pre-eclampsia as seen in our patient, are more frequent in patients with bladder extrophy is not known, but might be plausible and has been reported before [19]. Deans *et al.* reported a pre-eclampsia rate of 32% in a series of 57 pregnancies in 19 patients with bladder extrophy [8]. All pregnancies in patients with any abnormalities from the extrophy-epispadias-complex should therefore be classified as high risk pregnancies and should be followed at a perinatal center, where signs of growth restriction or placental insufficiency can be sufficiently assessed, and a surgical team which is able to perform a cesarean section in this complicated setting can be assembled at any time.

## Conclusion

As patients with bladder extrophy and other anomalies from the extrophy-epispadias-complex reach adolescence/adulthood, they need continuous medical follow-up and transition of care to adult surgery and gynecology in order to address specific aspects of sexual health, reproduction, contraception, and also cancer screening. In the presented case lack of transition of care resulted in an unplanned and complicated pregnancy.

## Data Availability

Not applicable.
